# URPD: a specific product primer design tool

**DOI:** 10.1186/1756-0500-5-306

**Published:** 2012-06-19

**Authors:** Li-Yeh Chuang, Yu-Huei Cheng, Cheng-Hong Yang

**Affiliations:** 1 Department of Chemical Engineering, Institute of Biotechnology and Chemical Engineering, I-Shou University, Kaohsiung, Taiwan; 2 Department of Digital Content Design and Management, Toko University, Chiayi, Taiwan; 3 Department of Electronic Engineering, National Kaohsiung University of Applied Sciences, Kaohsiung, Taiwan

**Keywords:** Polymerase chain reaction (PCR), Primer design, Web-based, Memetic algorithm (MA), Genetic algorithm (GA), Virtual gel electrophoresis

## Abstract

**Background:**

Polymerase chain reaction (PCR) plays an important role in molecular biology. Primer design fundamentally determines its results. Here, we present a currently available software that is not located in analyzing large sequence but used for a rather straight-forward way of visualizing the primer design process for infrequent users.

**Findings:**

URPD (yoUR Primer Design), a web-based specific product primer design tool, combines the NCBI Reference Sequences (RefSeq), UCSC In-Silico PCR, memetic algorithm (MA) and genetic algorithm (GA) primer design methods to obtain specific primer sets. A friendly user interface is accomplished by built-in parameter settings. The incorporated smooth pipeline operations effectively guide both occasional and advanced users. URPD contains an automated process, which produces feasible primer pairs that satisfy the specific needs of the experimental design with practical PCR amplifications. Visual virtual gel electrophoresis and in silico PCR provide a simulated PCR environment. The comparison of Practical gel electrophoresis comparison to virtual gel electrophoresis facilitates and verifies the PCR experiment. Wet-laboratory validation proved that the system provides feasible primers.

**Conclusions:**

URPD is a user-friendly tool that provides specific primer design results. The pipeline design path makes it easy to operate for beginners. URPD also provides a high throughput primer design function. Moreover, the advanced parameter settings assist sophisticated researchers in performing experiential PCR. Several novel functions, such as a nucleotide accession number template sequence input, local and global specificity estimation, primer pair redesign, user-interactive sequence scale selection, and virtual and practical PCR gel electrophoresis discrepancies have been developed and integrated into URPD. The URPD program is implemented in JAVA and freely available at http://bio.kuas.edu.tw/urpd/.

## **Findings**

### **Background**

PCR (polymerase chain reaction) is one of the most popular technologies used in biological and biomedical studies. It is a time-saving and sensitive method that amplifies specific regions of the genome. Primer design is of fundamental importance in PCR-based methods. Many parameters and aspects need to be taken into account when designing primers; the common ones are primer length/length difference, GC content, product size, the concentrations of the PCR buffer reagents, stable secondary structures, the melting temperature/melting temperature difference, and the nucleotide composition of the primer 3” end (i.e., GC clamp), etc. [1-4]. Furthermore, the analysis for template secondary structure, such as stem-loop structures in the template, particularly for RNA is advanced consideration on primer binding. Many methods have been developed to solve the inherent problems in primer design, e.g., genetic algorithms (GA) [5], greedy algorithms [6], and memetic algorithms (MA) [7]. Moreover, automated procedures are often implemented to facilitate the PCR experiment. Several web-based services are available that provide primer design. One example is the Primer3 software, which is a popular and frequently used primer design tool. Primer3 considers many different parameters, which allows users to reach their different objectives [8,9]. PrimerBlast, developed at NCBI, uses Primer3 to design primers; it provides a specificity check using BLAST to avoid causing amplification of targets other than the input template. In addition, UniPrime2 [10,11], Primer3Plus [12], and PDA [13], are also useful tools for primer design. In this study, we provide a new, user-friendly web interface primer design service called URPD (yoUR Primer Design), which combines the NCBI Reference Sequences (RefSeq), UCSC In-Silico PCR, memetic algorithm (MA) and genetic algorithm (GA) primer design methods [7] to obtain feasible primer sets.

### **Implementation**

#### *Primer design*

The MA and GA primer design methods are introduced into URPD as the core of the primer design. GA which is a well-known and heuristic search method that mimics the process of natural evolution [14-16] is inspired by Darwin's theory. In a GA, a solution is called an individual or a chromosome. A population contains many individuals, and each individual is different. The GA use evolutionary computations, i.e., selection, crossover, mutation and replacement, to generate new offsprings from generation to generation. After each generation, individuals in a GA share information with each other and the superior individuals based on a fitness rule are refined thus the optimal solution is found. MA is inspired by Dawkins” notion of a meme [17]. It is similar but superior to GA. MA progress through a local search before being involved in the evolution process [18]. An MA assures that all individuals and offsprings gain some experiences thus the optimal solution is obtained from generation to generation. Both MA and GA are based on the natural process of evolution through reproduction. The major difference between the MA and the GA is the local search mechanism in the MA, which improves its search results and reduces the likelihood of premature convergence encountered in the GA [7]. Some useful processes, such as reading the information from a file, output of results via network transmission, better run selection, and a primer ranking mechanism are integrated in the system. The fundamental performance (accuracy and run time) of the MA primer design was compared to a GA and is shown in [7]. Furthermore, we also introduce the thermodynamic nearest-neighbor model [19] to enhance the melting temperature calculation of the system.

#### *Sequence Databaseintegration*

The NCBI RefSeq [20] is integrated in URPD to provide available template sequences. By entering the Nucleotide Accession number, the corresponding sequence is obtained as the template sequence to perform the primer design. Moreover, UCSC InSilico PCR [21] is also integrated in URPD to acquire a virtual PCR product sequence from an available primer pair input as a new template sequence for redesigning the primer into more feasible ones. This allows users to perform an iterative design and modify potential PCR primers.

#### *Pipeline mechanism*

**Figure 1:**
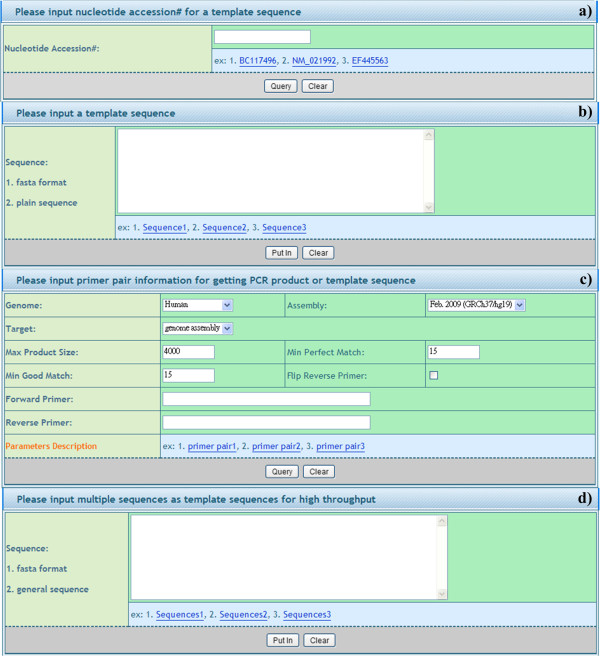
**Template sequence input.** Users can choose from four input interfaces, namely **a**) nucleotide accession number key, **b**) copying/pasting or manual typing a template sequence, **c**) primer pair information input, and **d**) copying/pasting or manual typing a template sequences for high throughput.

**Figure 2:**
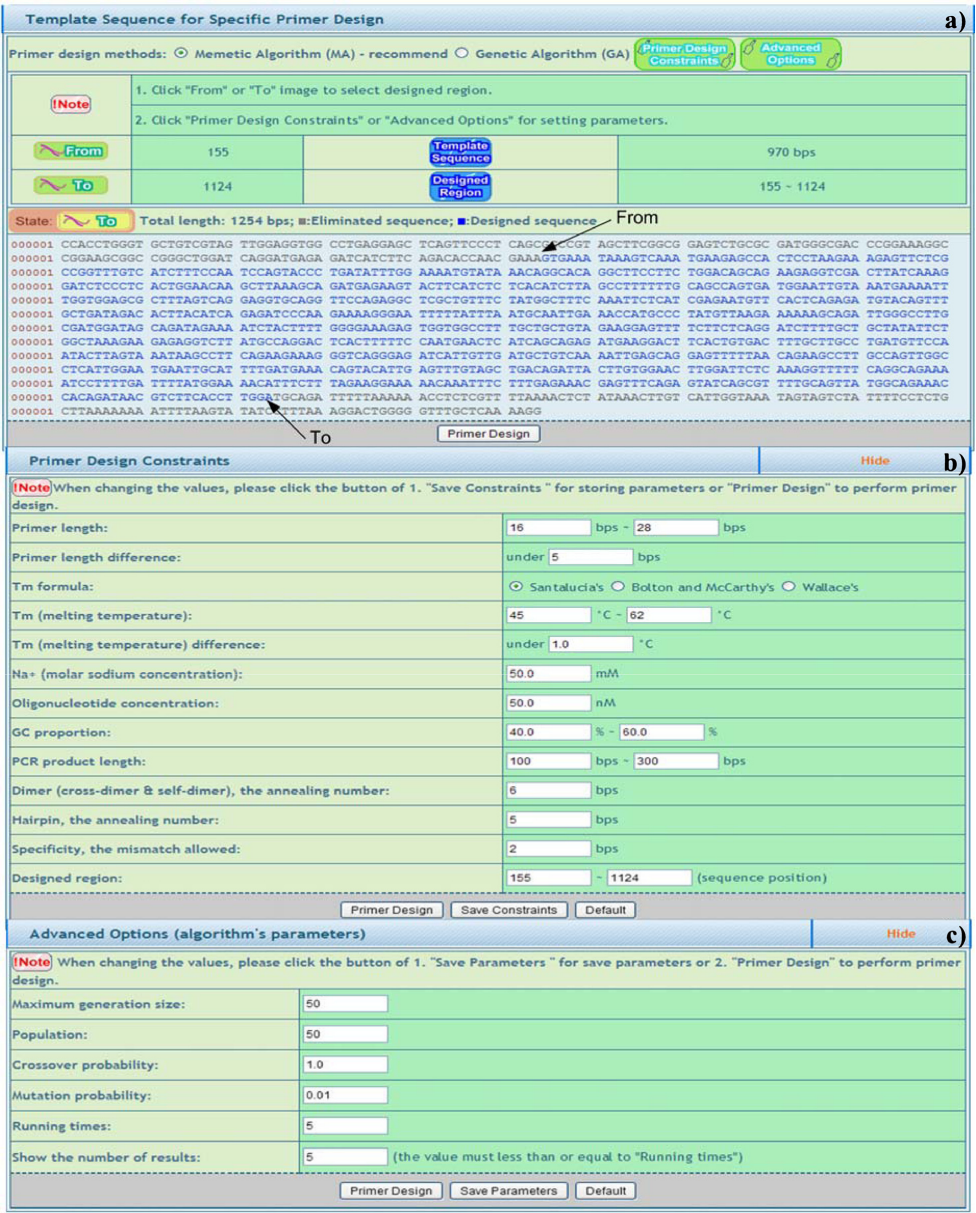
**Parameter settings.** The parameters have to be set via **a**) a selection of the sequence range (only for single template sequence, “From” and “To” are indicated by arrow lines), **b**) input of primer design constraints, and **c**) primer design algorithm parameter adjustment.

**Figure 3:**
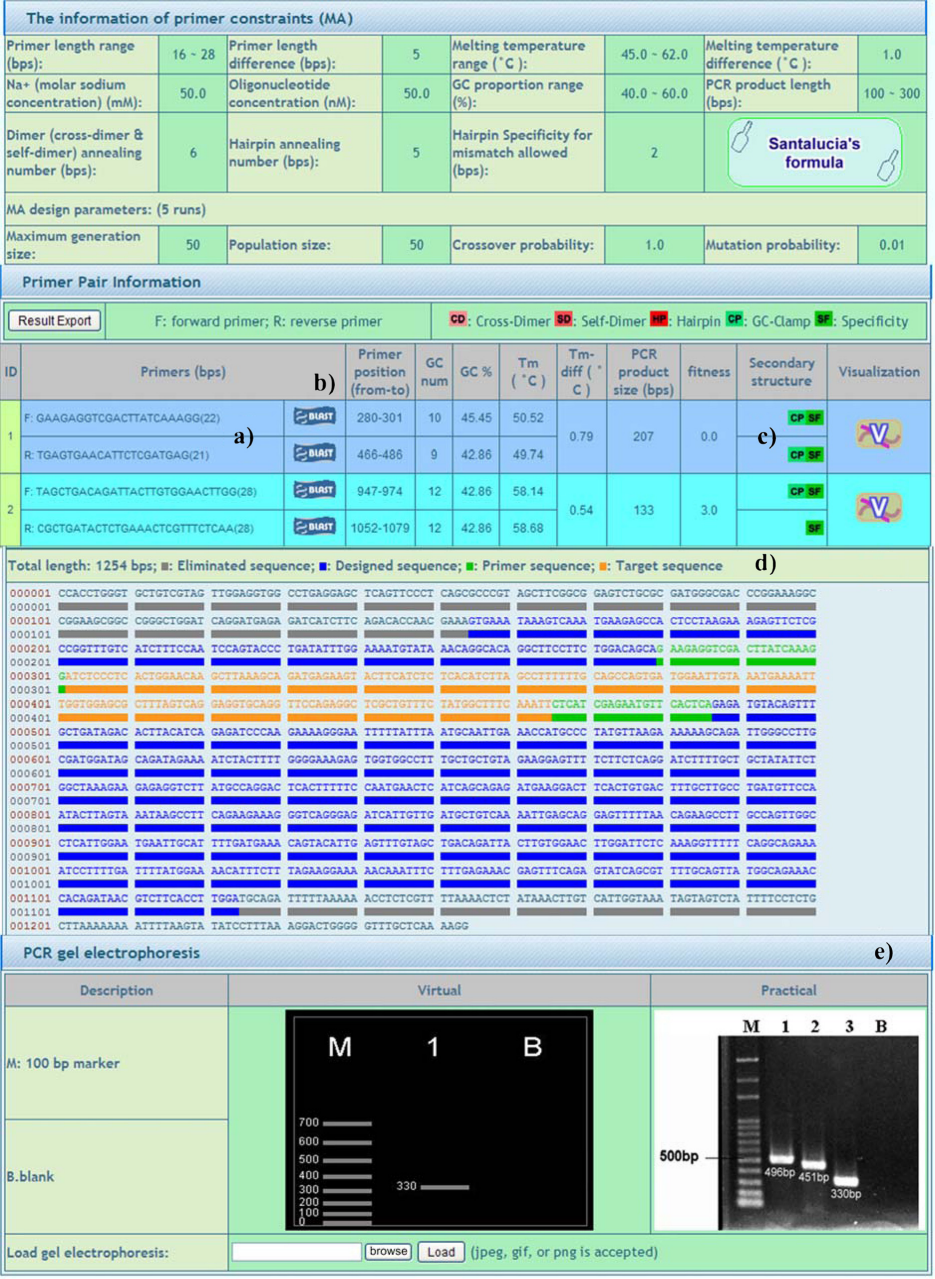
**Feasible primer pair output.** Feasible primer pairs are output: **a**) URPD provides a ranked primer pair output. The best primer set is always shown first. **b**) NCBI blast further enhances the specificity against the genome. **c**) Secondary structures of primers are clearly marked. **d**) The visualization shows the position of a primer pair and product information in a template sequence in color. **e**) A practical gel electrophoresis upload function is also available for comparison to virtual gel electrophoresis.

A pipeline mechanism is employed to effectively guide users. Three separate steps are included in the system. Template sequence input: users can choose from four input interfaces, namely a) nucleotide accession number key, b) copying/pasting or manual typing a template sequence, c) primer pair information input, and d) copying/pasting or manual typing a template sequences for high throughput (Figure 1). Secondly, the parameters can be set via a) a selection of a sequence range (only for single template sequence), b) input of primer design constraints, and c) primer design algorithm parameter adjustment (Figure 2). These parameters are useful for advanced users and are hidden by default for the occasional user. Finally, feasible primer pairs are output in one of four ways: a) URPD provides a ranked primer pair output (see Primer ranking mechanism for details). b) NCBI blast further enhances the specificity against the genome. c) Secondary structures of primers are clearly marked. d) The visualization shows the position of a primer pair and product information in a template sequence in color. e) A practical gel electrophoresis upload function is also available for comparison to virtual gel electrophoresis (Figure 3).

#### *Primer rankingmechanism*

URPD implements a reliable primer ranking mechanism, which outputs a ranking of feasible primer pairs based on both their fitness score estimated by the MA/GA primer design method and the melting temperature difference between the designed primer pairs. The best primer set is always shown first. The fitness score estimation is based on the primer constraints, which include the primer length, the primer length difference, the CG content, the melting temperature, the melting temperature difference, the PCR product size, dimers, hairpins, and the specificity (the detail estimation for fitness score is referred to [7]).

#### *Secondary structuremarkers*

Five secondary structures of primers are identified by clear markers in the URPD output. These are cross-dimers, self-dimers, hairpins, GC-clamps, and the specificity (Figure 3-c). This function is very useful for occasional users. It allows users to judge the quality of the designed primers. Cross-dimers, self-dimers, and hairpins are referred to AutoDimer [22]. GC-clamp checks whether the 3” terminal end of a primer is either nucleotide “G” or “C”. The check is used to avoid the denaturation of a primer when the temperature in the test tube is raised [9]. The specificity is evaluated for adequate primer annealing to the relevant positions by matching template sequences (internal) [23] and NCBI blast (external) [24]. Both the local and global searches are performed to further enhance the specificity.

#### *Designed regionselection andvisualization*

During the setting of parameters for the primer design, the designed region can be determined from a single template sequence by user system interaction (USI) (Figure 2-a). The function can be used to judge the quality of the input template sequence. After the primer design, the position of a primer pair and the product information in a template sequence is output visually (Figure 3-d). Via this function, users can explicitly identify the locations of primers and select primers suitable for their own experiments. Moreover, visual virtual gel electrophoresis provides a simulated PCR environment which compares results to those of practical gel electrophoresis (Figure 3-e). This provides a helpful validating mechanism in PCR experiments.

### **Results and discussions**

#### *Common outputof URPD*

Information regarding the primer constraints, such as primer length range, primer length difference, melting temperature range (°C), Na^+^(molar sodium concentration), GC proportion range (%), PCR product length (bps), dimer (cross-dimer & self-dimer) annealing number (bps), hairpin annealing number (bps), and specificity for mismatches allowed (bps) are commonly output. Algorithm design parameters, such as the maximum generation size, population size, crossover probability, and mutation probability are also shown. Finally, feasible primer information, such as primer length (bps), primer position (from-to), GC number, GC%, Tm (°C), Tm-diff (°C), PCR product size (bps), secondary structure, and visualization (Figure 3) are provided for all the inputs in URPD (Figure 1). The designed primer information can be output in a text file and then saved.

#### *Experimental validationfor URPDanalysis*

**Figure 4:**
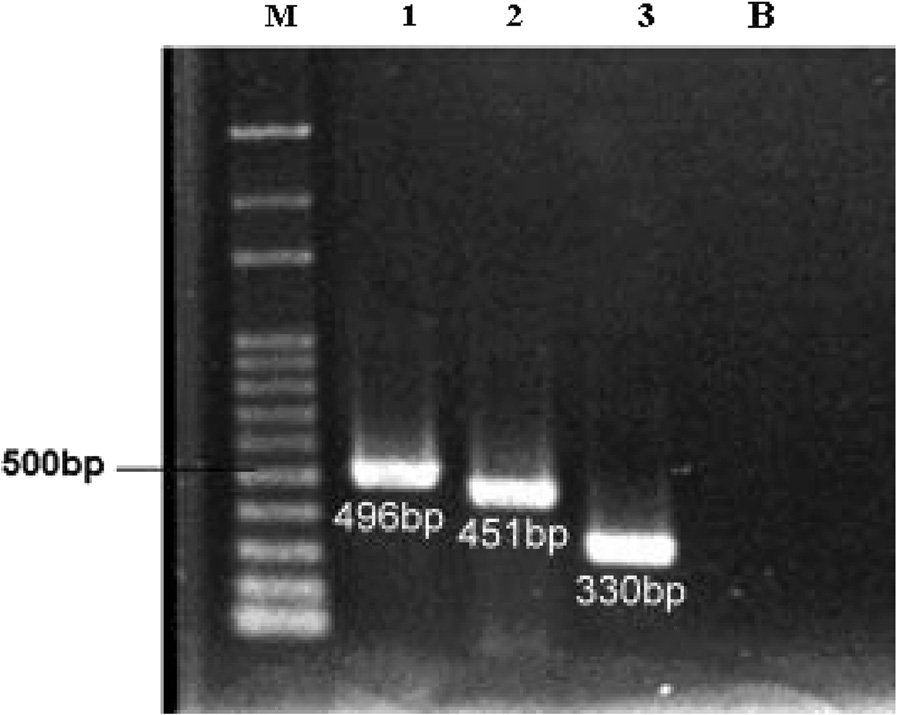
**Validation of the URPD system by PCR analysis of the blaOXA resistance gene.** Lane 1: 496 bp blaOXA amplified products for the blaOXA resistance gene. Lane 2: 451 bp blaOXA amplified products for the blaOXA resistance gene. Lane 3: 330 bp blaOXA amplified products for the penicillin-binding protein. The products were run on a 1%-agarose gel with a 100 bp ladder as a molecular size marker (Lane M)

Chromosomal DNAs of Acinetobacter baumannii ATCC 19606 and the Staphylococcus aureus strain were extracted by a recommended protocol from “Molecular Cloning: A Laboratory Manual” [25]. These two DNAs were used as templates for producing the OXA-98 beta-lactamase gene (blaOXA) and penicillin-binding protein (pbp) PCR analysis. The DNA templates used for verification of the oligonucleotide primers included the genes encoding β-lactamase gene (blaOXA 212 F and blaOXA 274 F) and penicillin binding protein gene (pbp 514 F). For all the genes, reaction mixtures (50 μL) included 2 μL template DNA (50 ng/μL), 5.0 μL of 10X Reaction Buffer (100 mM Tris–HCl , pH8.8 ; 500 mM KCl; 20 mM MgCl 2; 1% Triton X-100), 0.5 μL of YEAtaq DNA polymerase (5 U / μL), 1.0 μL of 10 mM dNTPs Mix, 10 pmol of each of the two primers, and 39.5 μL of sterile water. The PCR program had the following parameters: 94°C (10 min); 35 cycles at 94°C for (1 min), 55°C (1 min), 72°C (1 min); and 72°C (10 min). The presence and sizes of amplicons were assessed by electrophoresis in 1% agarose gels stained with 0.001 mg/mL ethidium bromide. The PCR gel electrophoresis is shown in Figure 4, and the primers designed using accession number HQ425495.1 and AF540028.1 for blaOXA and pbp, respectively, are listed in Table 1.

**Table 1:** - **Nucleotide Sequences of Oligonucleotides used for PCR Amplification**

**Species ID**	**Primers**	**Nucleotide sequence (5’-3’)**	**Primer length (bps)**	**Tm (°C)**	**GC%**	**PCR targets**	**Size (bp)**
ATCC 19606	*bla*_OXA212F_	GTGCTTCGACCGAGTATG	18	45.68	55.56	*bla*_OXA_ resistance gene	496
	*bla*_OXA707R_	ACAACCATCCAGTTAACC	18	41.12	44.44		
ATCC 19606	*bla*_OXA274F_	GAGCACCATAAGGCAACC	18	45.68	55.56	*bla*_OXA_ resistance gene	451
	*bla*_OXA724R_	TATTCCCTTGAGGCTGAAC	19	44.32	47.37		
ATCC 6538p	*pbp*_514F_	ATGCTTTACGACAAAGTTTC	20	41.25	35.00	penicillin-binding protein	330
	*pbp*_843R_	TCTCAGCTAACATGTATGC	19	42.17	42.11		

The primers designed using accession number HQ425495.1 and AF540028.1 for *bla*_OXA_ and *pbp*, respectively. F and R under the primers field represent forward primer and reverse primer, respectively. The number under the primers field represents the exact placement of the primer within the target sequence, e.g., the 212 F represents that the forward primer has the exact placement of 212 within the target sequence.

#### *Comparison toother primerdesign software*

To date, various tools for facilitating primer design have been developed. Primer3Plus is the web based interface of the popular Primer3 primer design program and constitutes a superior alternative to the CGI-scripts that come with Primer3 [12]. UniPrime2 retrieves and aligns homologous sequences from Genbank automatically, identifies regions of conservation within the alignment, and generates suitable primers that allow amplification of various genomic regions [11]. PDA is a web-based tool that uses thermodynamic theories to evaluate the fitness of primers for primer design [13]. We compared URPD with these three web-based primer design software tools, i.e., Primer3Plus, UniPrime2, and PDA, in Table 2. We found that URPD is suitable for both advanced and occasional users and can facilitate small-scale experiments in regular laboratories.

**Table 2:** Comparison of URPD with Primer3Plus, UniPrime2, and PDA

**Features**	**Software**	**URPD**	**Primer3Plus**	**UniPrime2**	**PDA**
Type		web-based	web-based	web-based	web-based
Availability		http://bio.kuas.edu.tw/urpd/	http://www.bioinformatics.nl/primer3plus/	http://uniprime.batlab.eu/	http://dbb.nhri.org.tw/primer/
Primer design method		Memetic Algorithm (MA) & Genetic Algorithm (GA)	Primer3-based	Primer3-based	✘
User Interface		intuitive user interface	intuitive user interface, but complicated parameter settings	intuitive user interface	brief user interface
Sequence import		single sequence or multiple sequences (high throughput)	single sequence	single sequence	single sequence or multiple sequences (high throughput)
Accession No. input		✓	✘	✘	✘
UCSC In-silico PCR		✓	✘	✘	✘
NCBI blast check		built-in	hyperlink	built-in	✘
Template annealing check		✓	✘	✘	✘
Pipeline leader		✓	✘	✓	✓
Ranking mechanism		✓	✘	✘	✓
Result file export		text file	text file	e-mail	MS-Excel
Visual PCR (gel electrophoresis)		✓	✘	✘	✘

Note: Symbol ✓ indicates that this function is included; symbol ✘ indicates that this function is not available.

#### *Contributions ofURPD*

URPD's major contribution to primer design is the fact that it provides a method for reliably and simultaneously evaluating the many constraints involved, and thus allows users to better screen primers at each of iteration. URPD uses heuristic methods to find possible primers and is thus feasible for the analysis of large quantities of DNA templates. The contributions of URPD to primer design are summarized below:

1) The friendly and graphic user interface with its built-in parameters and incorporated smooth pipeline operations guides users in obtaining the desired results effectively. It also enables infrequent users to easily operate the system.

2) URPD contains an automated process, which produces feasible and specific primer pairs that satisfy the specific needs of the experimental design.

3) Accession numbers contained in NCBI RefSeq for different organisms are available as template sequences for the design of feasible primers.

4) URPD provides a high throughput primer design function that is useful for large-scale experiments.

5) UCSC In-Silico PCR is integrated in URPD to acquire the product sequences that can be used to redesign the primers into more feasible ones (i.e., modification of potential PCR primers).

6) URPD contains a reliable primer ranking mechanism, outputs feasible primer set rankings based on both a fitness score estimated by effective methods and the melting temperature difference between designed primer pairs.

7) Visual virtual gel electrophoresis and in-silico PCR provide a simulated PCR environment.

8) Comparison of practical gel electrophoresis and virtual gel electrophoresis facilitates the PCR experiment.

### **Conclusions**

URPD has been applied to design primers and subsequently used to perform PCR experiments successfully. It is a user-friendly tool that provides specific primer design results. The pipeline design path allows easy operation, even by a beginner. Single template sequence and high throughput primer design are provided in URPD.

Moreover, advanced parameter settings can be accessed and set for more sophisticated PCR experiments. Several significantly novel functions, such as a nucleotide accession number template sequence input, local and global specificity estimation, primer pair redesign, user-interactive sequence scale selection, and virtual and practical PCR gel electrophoresis discrepancies have been developed and integrated into URPD.

### **Availability and requirements**

The URPD web site and its user manual are freely accessible at http://bio.kuas.edu.tw/urpd and http://bio.kuas.edu.tw/urpd/userManual.jsp, respectively. The user manual is also inculded as Additional file 1. The feasible primers are analyzed on-line. Both NCBI RefSeq [20] and UCSC In-Silico PCR [21] are constantly updated and retrieved on-line in an automated procedure. Our proposed application is mainly based on the iterative parameters of the population size and the running times. By setting the iterative parameters to smaller values, a faster performance is achieved; however, this impacts the quality of the designed parameters negatively. The default values, i.e., a population size of 50 and five test runs, are usually sufficient for general primer designs. The retrieval formats for the used on-line databases is checked monthly to maintain proper on-line extraction of data.

Project name: URPD: A Specific Product Primer Design Tool.

Project home page: http://bio.kuas.edu.tw/urpd/.

Operating system(s): Operating systems free with web browser. Programming language: Java.

Other requirements: Java 1.6.

License: none for academic users. For any restrictions applying to the use by nonacademics please contact the corresponding author.

## **Availability of supporting data**

The data supporting the results of this article are included within the article (as Table 1). The data supporting the results of this article are also available at http://bio.kuas.edu.tw/urpd/experiments.jsp.

## Abbreviations

blaOXA: OXA-98 beta-lactamase gene; CGI: Common Gateway Interface; bps: base pairs; DNA: Deoxyribonucleic Acid; GA: Genetic Algorithm; MA: Memetic Algorithm; pbp: penicillin-binding protein; PCR: Polymerase Chain Reaction; zPDA: Primer Design Assist; RefSeq: NCBI Reference Sequences; Tm: Melting Temperature; URPD: YoUR Primer Design; USI: User System Interaction.

## Competing interests

The authors declare they have no competing interests.

## Authors’ contributions

L-YC provided the biochemistry background, introduced the bioinformatics needed, and verified the PCR experiment. Y-HC participated in the design of the algorithm, the development of the system, and the writing of the manuscript. C-HY coordinated and oversaw this study, and modified the manuscript. All authors read and approved the final manuscript.

## Supplementary Material

Additional file 1 The user manual for URPD (yoUR Primer Design) - A Specific Product Primer Design Tool. Click here for file
