# Supplementary material for: URPD: a specific product primer design tool
Source: BMC Res Notes. 2012 Jun 19;5:306. doi: 10.1186/1756-0500-5-306 (PMC3494561; doi:10.1186/1756-0500-5-306)
Supplement: Additional file 1 — The user manual for URPD (yoUR Primer Design) - A Specific Product Primer Design Tool. [file 1756-0500-5-306-S1.pdf]

URPD (yoUR Primer Design)

A Specific Product Primer Design Tool

User Manual v1.0

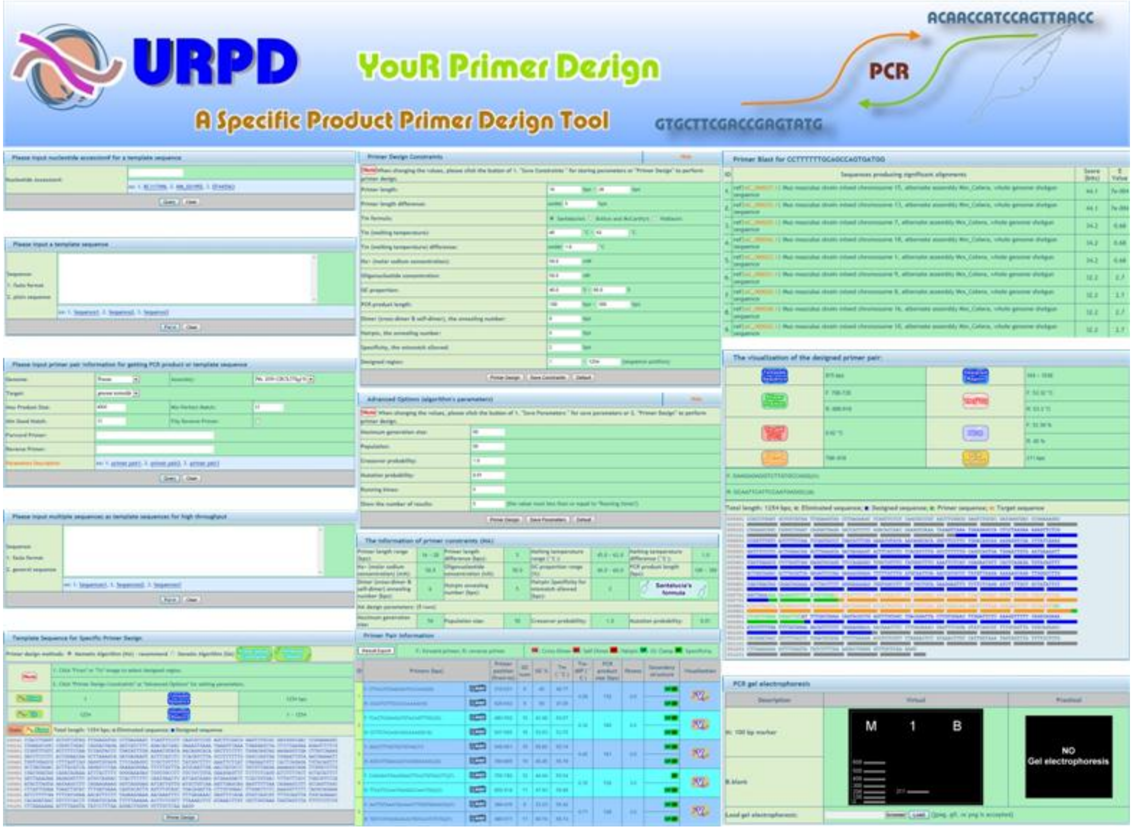

Contact Authors:

Li-Yeh Chuang: chuang@isu.edu.tw
Yu-Huei Cheng: yuhuei.cheng@gmail.com
Cheng-Hong Yang: chyang@cc.kuas.edu.tw

Updated on March 22, 2012

Table of contents

1. Introduction..2

2. Template Sequence Input..3

2.1. Nucleotide Accession# key in..3

2.2. Template sequence input...4

2.3. Primer pair information input...4

2.4. Copying and pasting template sequences for high throughput...........6

3. Parameter Settings...7

3.1. Sequence range selection to trim the template sequence....7

3.2. Setting of primer design constraints and primer design algorithm parameters..........9

4. Feasible Primer Pairs...11

4.1. Blast specific estimation...13

4.2. Secondary structures..13

4.3. Visualization..14

4.4. Result file output..16

1. Introduction

URPD (yoUR Primer Design), a web-based primer design tool, combines the NCBI Reference Sequences (RefSeq), UCSC In-Silico PCR, memetic algorithm (MA) and genetic algorithm (GA) primer design methods to obtain specific primer sets. A friendly user interface is accomplished by built-in parameters setting. The incorporated pipeline operations effectively guide both, advanced and occasional users. URPD contains an automated process which produces feasible primer pairs that satisfy the specific needs of the experimental design with practical PCR amplifications. Visual virtual gel electrophoresis and *in-silico* PCR provide a simulated PCR environment. A comparison of practical gel electrophoresis with virtual gel electrophoresis is used to validate the PCR experiment. Wet-laboratory validation proved that the system provides feasible primers. URPD is freely available at http://bio.kuas.edu.tw/urpd/.

URPD designs specific primer sets in three steps. The first step is the template sequence input; followed by the parameter settings. In a third step, feasible primer pairs are output. Figure 1 illustrates the three steps, respectively. The following sections describes the further processes of the pipeline.

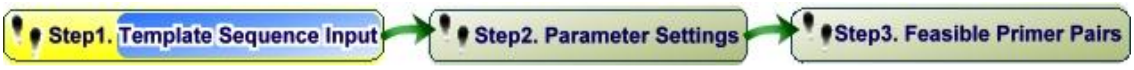

Figure 1. Primer design with URPD.

2. Template Sequence Input

Four input types are available in URPD: *2.1. Nucleotide Accession# key in*; *2.2. Template sequence input*; *2.3. Primer pair information input;* and *2.4. Coping and pasting template sequences for high throughput*. These steps are illustrated below.

2.1. Nucleotide Accession# key in

The NCBI Reference Sequences (RefSeq) provides a non-redundant collection, which includes sequences from plasmids, organelles, viruses, archaea, bacteria, and eukaryotes. It contains richly annotated DNA, RNA, and protein sequences from diverse taxa. URPD, in combination with NCBI Reference Sequences (RefSeq), provides a comprehensive, standard template sequence to design primers. All Nucleotide Accession# available in RefSeq are also available in URPD. Figure 2 shows the interface for Nucleotide Accession# input.

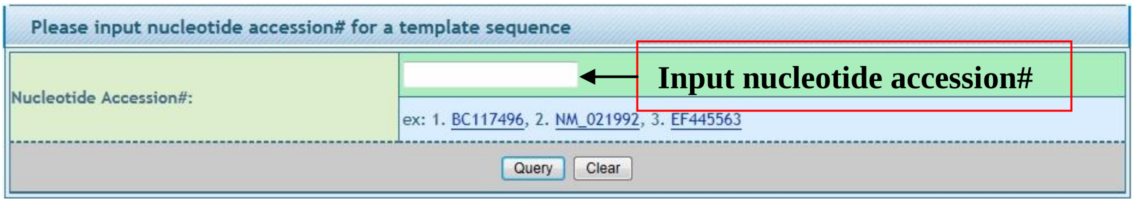

Figure 2. Nucleotide Accession# input interface.

2.2. Template sequence input

A template sequence with FASTA format or plain format can be used to enter the template sequence. It is a universal function that exists in many primer design tools and is suitable for a small-scale experiment. Figure 3 shows the template sequence paste input interface.

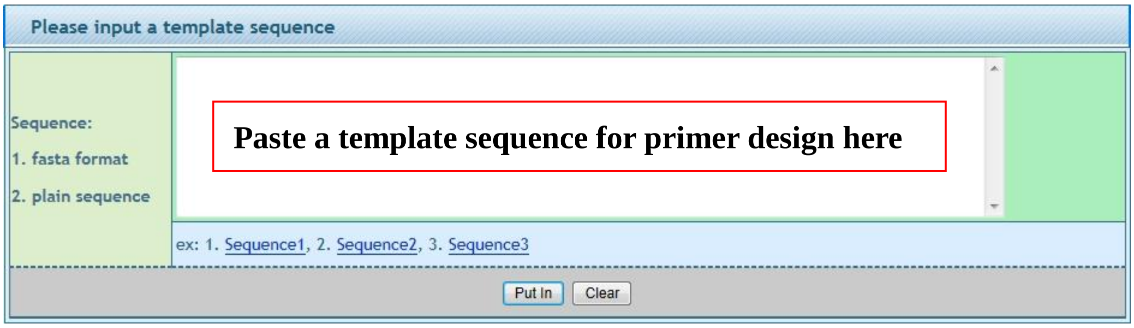

Figure 3. Template sequence input interface.

2.3. Primer pair information input

Many primers designed by other primer design tools are inadequately annealed to irrelevant positions. UCSC In-Silico PCR effectively searches a sequence database for a pair of PCR primers to confirm their specificity. Furthermore, an unknown template sequence can be retrieved by a primer pair and different primer pairs can be designed. Figure 4 shows the primer pair information input interface, and Figure 5 shows the parameter descriptions of UCSC In-Silico PCR, which can be opened by clicking on the 'Parameters Description' hyperlink.

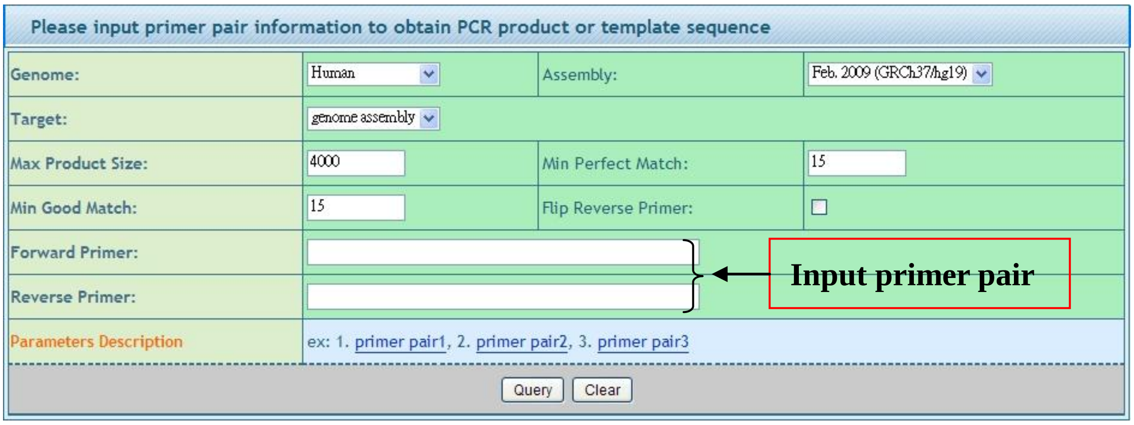

Figure 4. Primer pair information input interface.

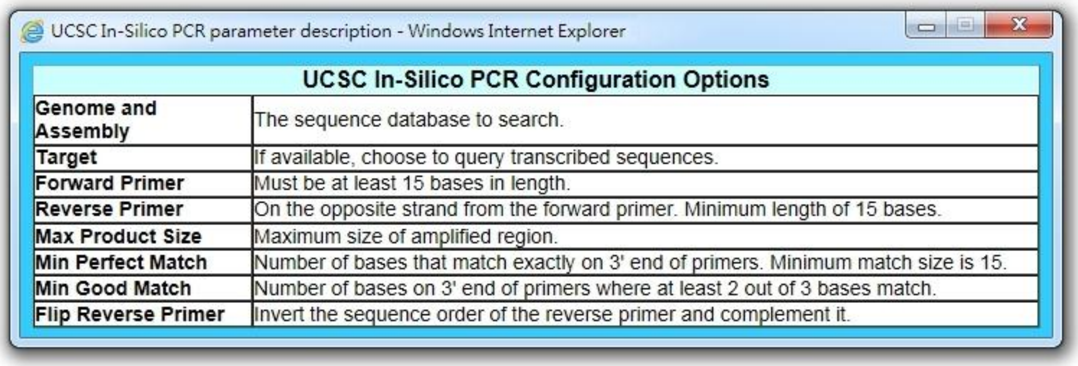

UCSC In-Silico PCR parameter description - Windows Internet Explorer

UCSC In-Silico PCR Configuration Options	
**Genome and Assembly**	The sequence database to search.
**Target**	If available, choose to query transcribed sequences.
**Forward Primer**	Must be at least 15 bases in length.
**Reverse Primer**	On the opposite strand from the forward primer. Minimum length of 15 bases.
**Max Product Size**	Maximum size of amplified region.
**Min Perfect Match**	Number of bases that match exactly on 3' end of primers. Minimum match size is 15.
**Min Good Match**	Number of bases on 3' end of primers where at least 2 out of 3 bases match.
**Flip Reverse Primer**	Invert the sequence order of the reverse primer and complement it.

Figure 5. UCSC In-Silico PCR parameter description.

2.4. Copy/paste template sequences for high throughput

Many template sequences with FASTA format or plain format can be pasted into the template sequences paste input mask. This is suitable for a large-scale experiment with high throughput. Figure 6 shows the template sequences input interface.

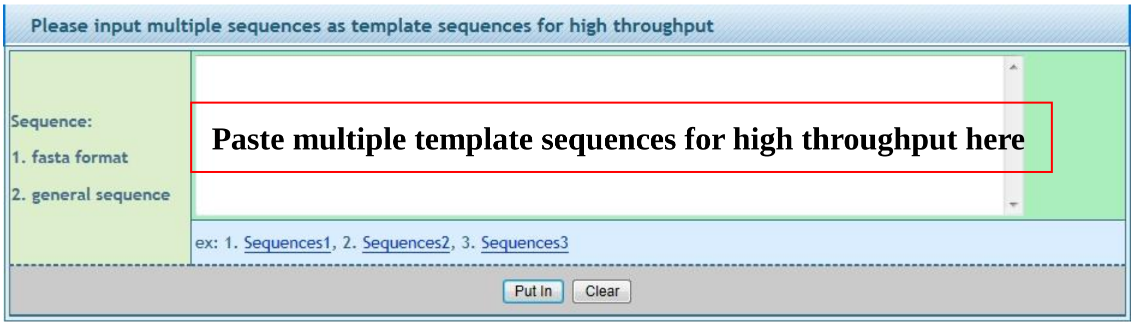

Figure 6. Input interface for high throughput template sequences.

3. Parameter Settings

After a template sequence has been imported into URPD, one of two specific primer design methods can be selected, i.e., a memetic algorithm (MA) or a genetic algorithm (GA) (Figure 7). The MA method is the recommended method for specific primer design in the system as it has been proven better than the GA method. URPD provides a sequence range selection that allows the template sequence to be trimmed (see ***3.1. Sequence range selection to trim the template sequence***). Primer design constraints can be individually set to allow for a more flexible experiential PCR experiment; the primer design algorithm parameters can also be adjusted to improve the primers qualities (see ***3.2. Setting of primer design constraints and primer design algorithm parameters***).

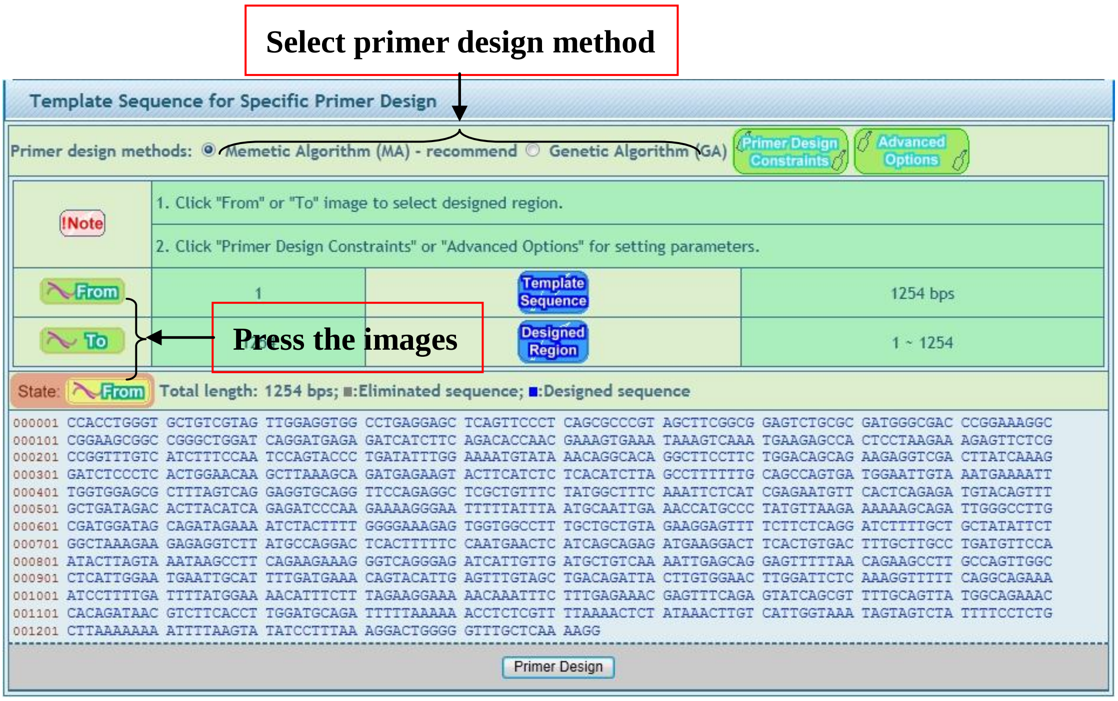

Figure 7. Importing a template sequence is imported to URPD.

3.1. Sequence range selection to trim the template sequence

When the image 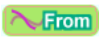

is pressed and the designed sequence in blue color is selected in Figure 7 as the start site, the trimmed sequence before the start site is displayed in gray color (Figure 8). By pressing the image 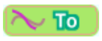

and then clicking on the designed sequence in blue color in Figure 7 as the end site, the trimmed sequence after the end site is displayed in gray color (Figure 8). Users can select the start and the end sites via the above operations to preserve the desired sequence for their primer design. Figure 8 shows the result of the sequence range selection.

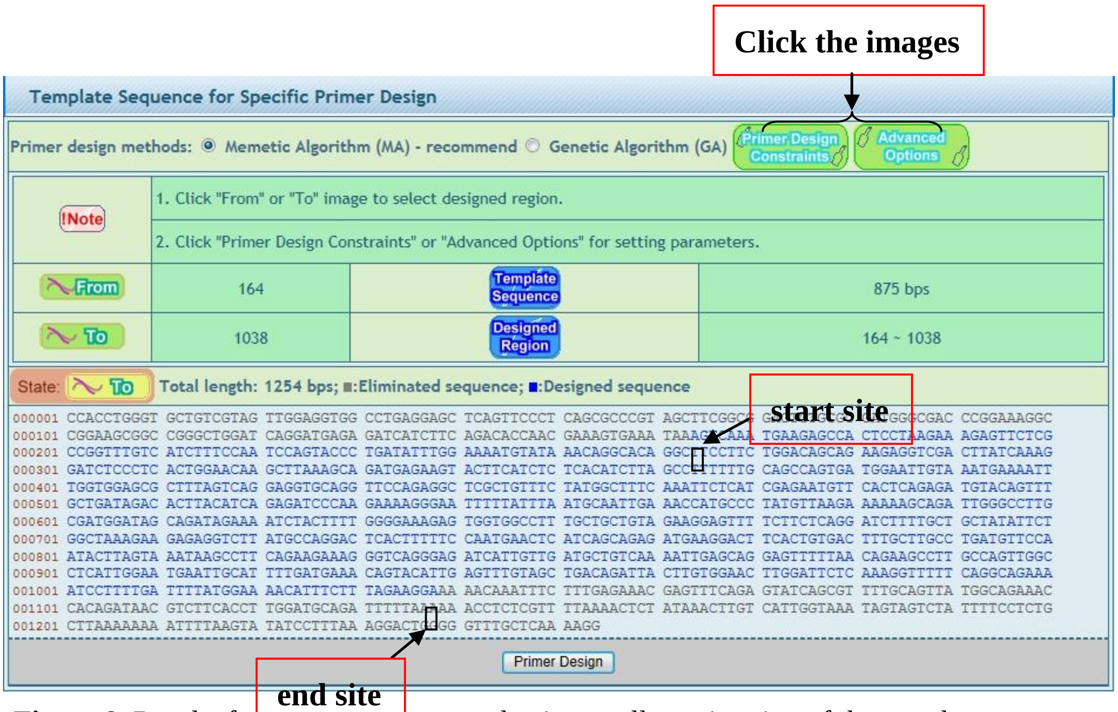

Figure 8. Results for a sequence range selection to allow trimming of the template sequence.

3.2. Setting of primer design constraints and primer design algorithm parameters

Advanced user can individually set the primer design constraints or primer design algorithm parameters by clicking the image 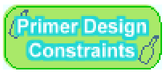

or 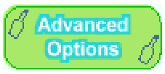

in Figure 8. Figure 9 shows the interface for the primer design constraints, and Figure 10 shows the interface for the primer design algorithm parameters. Primer design constraints in URPD are the primer length, the primer length difference, the Tm formula, the Tm (melting temperature), the Tm difference, the molar sodium (Na^{+}) concentration, the oligonucleotide concentration, the GC proportion, the PCR product length, the annealing number for a dimer (cross-dimer & self-dimer), the annealing number for hairpins, the mismatches allowed for specificity, and a manual region selection. Primer design algorithm parameters in URPD include the maximum generation size, the population size, the crossover probability, the mutation probability, the run time, and the number of results.

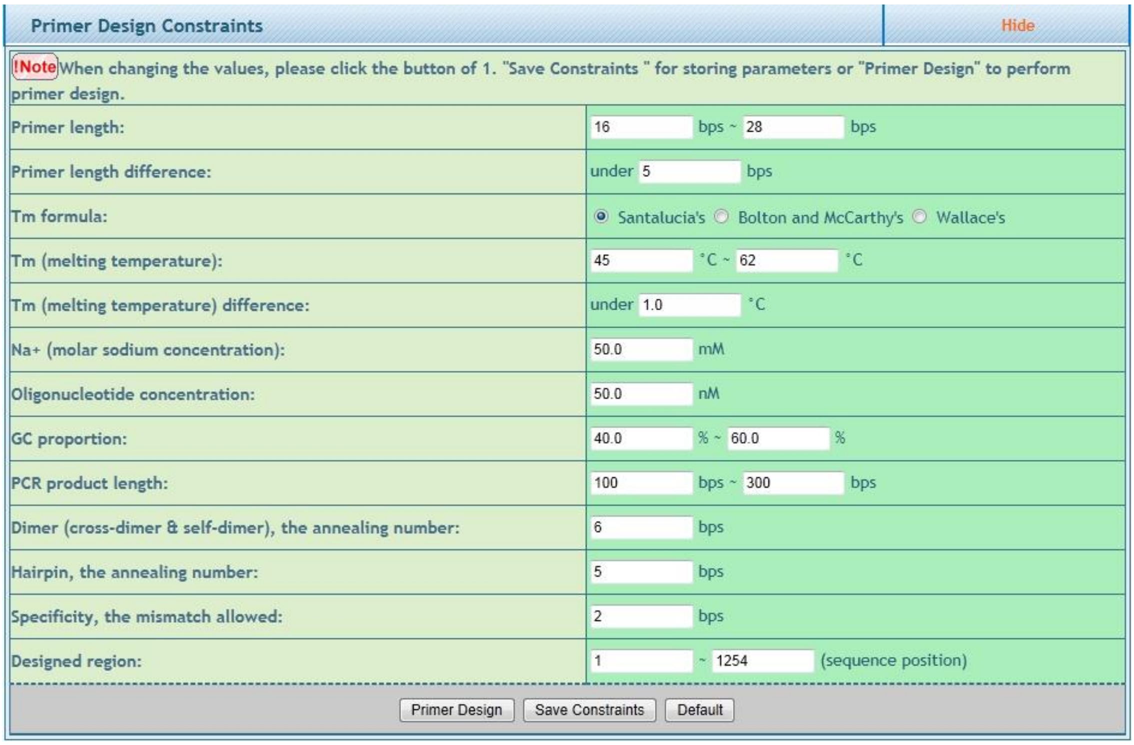

Figure 9. Interface showing the primer design constraints.

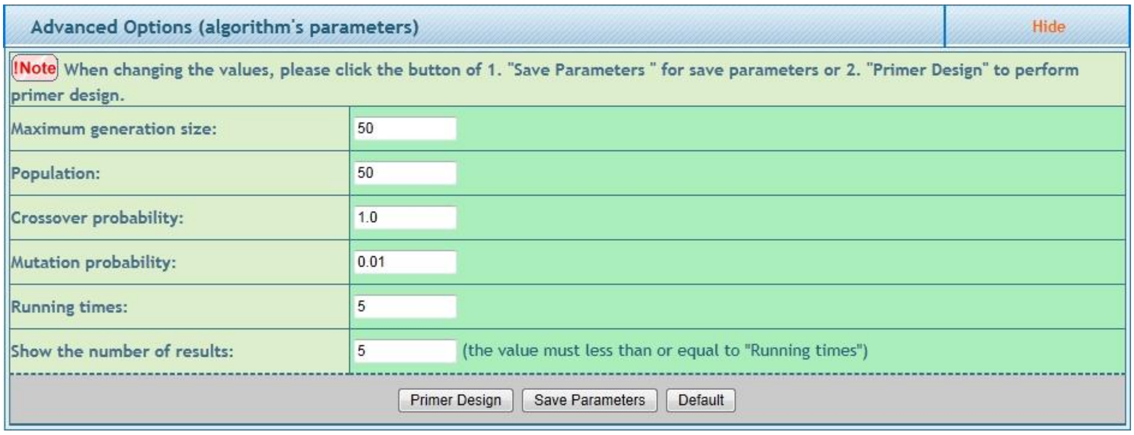

Figure 10. Interface showing the primer design algorithm parameters.

4. Feasible Primer Pairs

URPD provides an ordered primer pair output. The primer sets are ranked according to both their fitness value estimated by MA/GA primer design method and the melting temperature difference between designed primer pairs. The best primer pairs are always shown first. The primer pair information is comprised of the forward and reverse primers, blast specific estimation, primer position (from-to), the GC number, the GC%, the Tm (°C), the Tm-diff (°C), the PCR product size, secondary structures, and visualization. Secondary structures are marked by clear symbols and include cross-dimers (CD), self-dimers (SD), hairpins (HP), GC-clamp (CP), and the specificity (SF) (see ***4.2. Secondary structures***). The visualization shows the position of primer pairs and product information in a template sequence in color (see ***4.3. Visualization***). Figure 11 shows result for a URPD primer design.

Primer constraints information (MA)							
Primer length range (bps):	16 ~ 28	Primer length difference (bps):	5	Melting temperature range (˚C):	45.0 ~ 62.0	Melting temperature difference (˚C):	1.0
Na+ (molar sodium concentration) (mM):	50.0	Oligonucleotide concentration (nM):	50.0	GC proportion range (%):	40.0 ~ 60.0	PCR product length (bps):	100 ~ 300
Dimer (cross-dimer & self-dimer) annealing number (bps):	6	Hairpin annealing number (bps):	5	Hairpin Specificity for mismatch allowed (bps):	2	Santalucia's formula	
MA design parameters: (5 runs)							
Maximum generation size:	50	Population size:	50	Crossover probability:	1.0	Mutation probability:	0.01

Primer Pair Information

Export Results | F: forward primer; R: reverse primer | CD: Cross-Dimer SD: Self-Dimer HP: Hairpin CP: GC-Clamp SF: Specificity

ID	Primers (bps)		Primer position (from-to)	GC num	GC %	Tm (˚C)	Tm-diff (˚C)	PCR product size (bps)	fitness	Secondary structure	Visualization
1	F: TCAGGAGGTGCAGGTTCCAG(20)	BLAST	417-436	12	60	57.26	0.29	231	0.0	CP SF	V
	R: GCCACCACTCTTTCCCCAAAAG(22)	BLAST	626-647	12	54.55	57.55				CP SF	
2	F: TACTTTTGGGGAAAGAGTGGTG(22)	BLAST	624-645	10	45.45	52.24	0.36	207	3.0	CP SF	V
	R: CTTTCTTCTGAAGGCTTATTTACTAAG(27)	BLAST	804-830	9	33.33	51.88				CP SF	
3	F: TGGCCTCATTGGAATG(16)	BLAST	897-912	8	50	46.83	0.68	167	3.0	CP SF	V
	R: CTCGTTTCTCAAAGAAATTTG(21)	BLAST	1043-1063	7	33.33	46.15				CP SF	
4	F: TTCTGGCTAAAGAAGAGAGGTCTTATG(27)	BLAST	697-723	11	40.74	55.6	0.76	274	3.0	CP SF	V
	R: GTTCCACAAGTAATCTGTCAGCTACA(26)	BLAST	945-970	11	42.31	56.36				SF	
5	F: CTTTAGTCAGGAGGTGCAGGTTC(23)	BLAST	411-433	12	52.17	56.44	0.88	295	3.0	CP SF	V
	R: TAGCCAGAATATAGCAGCAAAAGATC(26)	BLAST	680-705	10	38.46	57.33				CP SF	

Figure 11. Result for a URPD primer design.

4.1. Blast specific estimation

NCBI blast is used to further estimate the specificity of the designed primers via a genomic sequence database. By clicking on the BLAST icon in Figure 11, URPD performs a blast search to confirm the specificity of the primer. Figure 12 shows blast results for a designed primer.

Primer Blast for CCTTTTTTGCAGCCAGTGATGG

ID	Sequences producing significant alignments	Score (bits)	E Value
1.	ref|AC_000037.1| Mus musculus strain mixed chromosome 15, alternate assembly Mm_Celera, whole genome shotgun sequence	44.1	7e-004
2.	ref|AC_000035.1| Mus musculus strain mixed chromosome 13, alternate assembly Mm_Celera, whole genome shotgun sequence	44.1	7e-004
3.	ref|AC_000029.1| Mus musculus strain mixed chromosome 7, alternate assembly Mm_Celera, whole genome shotgun sequence	34.2	0.68
4.	ref|AC_000040.1| Mus musculus strain mixed chromosome 18, alternate assembly Mm_Celera, whole genome shotgun sequence	34.2	0.68
5.	ref|AC_000023.1| Mus musculus strain mixed chromosome 1, alternate assembly Mm_Celera, whole genome shotgun sequence	34.2	0.68
6.	ref|AC_000031.1| Mus musculus strain mixed chromosome 9, alternate assembly Mm_Celera, whole genome shotgun sequence	32.2	2.7
7.	ref|AC_000030.1| Mus musculus strain mixed chromosome 8, alternate assembly Mm_Celera, whole genome shotgun sequence	32.2	2.7
8.	ref|AC_000038.1| Mus musculus strain mixed chromosome 16, alternate assembly Mm_Celera, whole genome shotgun sequence	32.2	2.7
9.	ref|AC_000032.1| Mus musculus strain mixed chromosome 10, alternate assembly Mm_Celera, whole genome shotgun sequence	32.2	2.7

Figure 12. Blast results for a designed primer.

4.2. Secondary structures

The secondary structures are marked by clear symbols. Cross-dimers are marked by the symbol CD; self-dimers are marked by the SD symbol; hairpins are marked by HP, GC-clamps are marked by CP, and the specificity is indicated by the SF symbol. The information is shown after a designed primer. Figure 11 shows that all designed primers are GC-clamps and specific. The symbols facilitate the evaluation of the primer quality for a user.

4.3. Visualization

URPD visually depicts the relationship between a designed primer set and a template sequence. By clicking on the 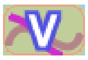 symbol, URPD opens a page that visualizes the results for a primer pair. Figure 13 shows eliminated sequences indicated by a gray bar. Template sequence are indicated by a blue bar, the designed primer pair is indicated by a green bar, and the target sequence is indicated by an orange bar. The designed primer pair information is listed on the left of the visualization.

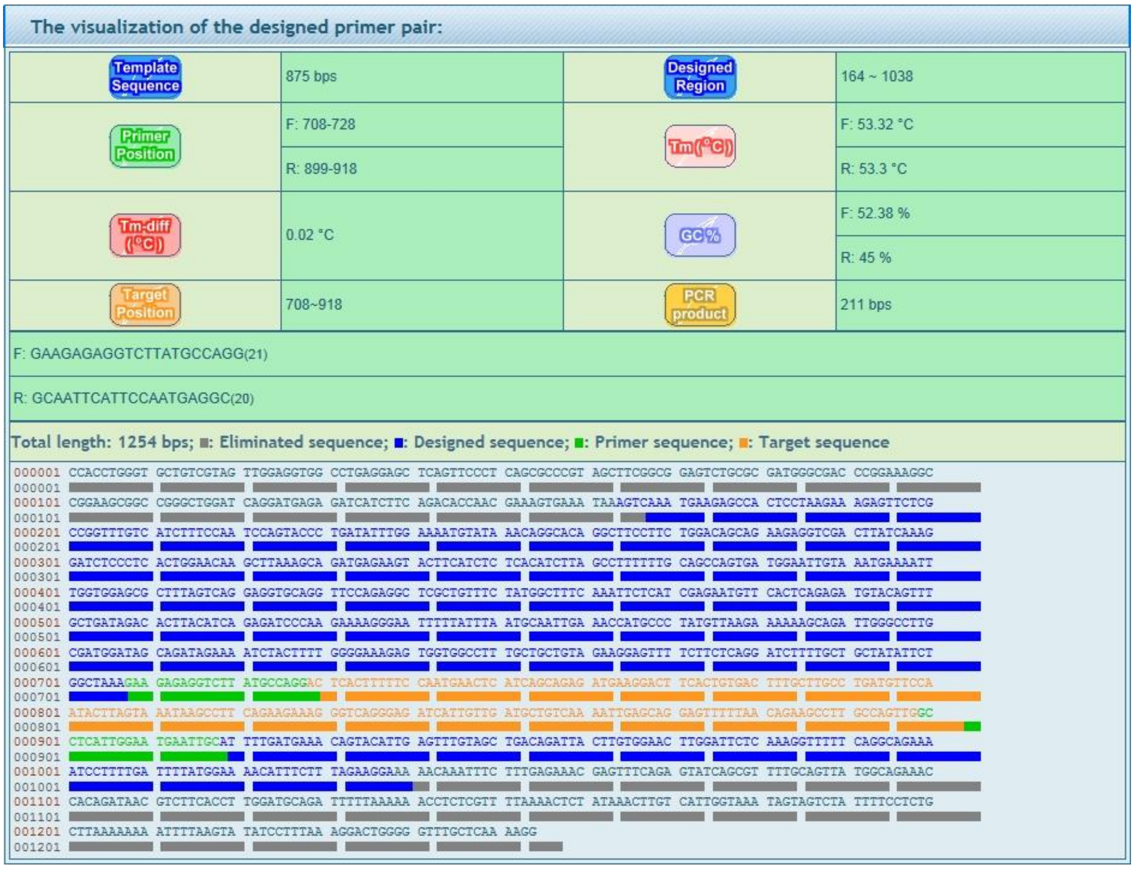

Figure 13. Sequence visualization provides the relevant primer pair information.

A virtual PCR gel electrophoresis is also generated by URPD and practical PCR gel electrophoresis can be loaded into URPD (Figure 14). The virtual PCR gel electrophoresis shows the product size of the designed primer pair. This allows a user to intuitively gauge the results of the PCR experiment. At the same time, users can load their practical PCR gel electrophoresis results and compare them with the virtual PCR gel electrophoresis results for further validation of the PCR experiment.

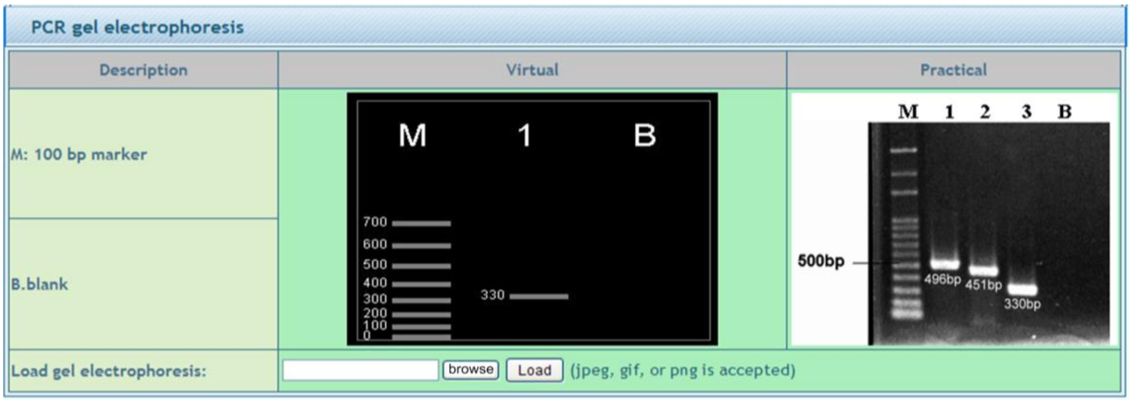

Figure 14. Virtual and practical PCR gel electrophoresis results.

4.4. Result file output

URPD provides an output of the results through in a text format file. In Figure 11, users can click on the “Export Results” button to perform this action. The output file contains sequence information, primer design constraints, algorithm parameters, and primer pair information, all of which are shown in Figure 15 to Figure **18**, respectively.

```
=============================== sequence information ===============================
Sequence data: CCACCTGGGTGCTGTCGTAGTTGGAGGTGGCCTGAGGAGCTCAGTTCCCTCAGCGCCCGTAGCTTCGGCGGAGTC
ATTTTATGGAAAACATTTCTTTAGAAGGAAAAACAAATTTCTTTGAGAAACGAGTTTCAGAGTATCAGCGTTTTGCAGTTATGGCAGAAA
Template DNA: CCACCTGGGTGCTGTCGTAGTTGGAGGTGGCCTGAGGAGCTCAGTTCCCTCAGCGCCCGTAGCTTCGGCGGAGTCT
TTTTATGGAAAACATTTCTTTAGAAGGAAAAACAAATTTCTTTGAGAAACGAGTTTCAGAGTATCAGCGTTTTGCAGTTATGGCAGAAAC
```

Figure 15. Sequence information retrieved from the output file.

```
=============================== primer constraints ===============================
Primer length (bps): 16 ~ 28
Primer length difference (bps): 5
Melting Tm (oC): 45.0 ~ 62.0
Melting Tm difference (oC): 5.0
Na+ (M): 0.5
GC proportion (%): 40.0 ~ 60.0
PCR product length (bps): 495 ~ 500
Dimer (cross-dimer & self-dimer) annealing number (bps): 6
Hairpin annealing number (bps): 5
Specificity for mismatch allowed (bps): 2
Tm formula: Wallace's formula
```

Figure 16. Primer constraints retrieved from the output file.

```
=============================== MA parameters ===============================
Maximum generation size: 1000
Population size: 100
Crossover probability: 1.0
Mutation probability: 0.01
Running times: 5
```

Figure 17. Algorithm parameters retrieved from the output file. The MA algorithm was used here.

```
====================== primer pair information:1 =======================
Optimal solution: (397, 21, 497, 22)
fitness_best: 0.0
Forward Primer: ATTTGGTGGAGCGCTTTAGTC
Forward Primer position: 397-417
Forward Primer GC number: 10
Forward Primer GC%: 47.61904761904761
Forward Primer length: 21
Reverse Primer: TGGCAAGGCTTCTGTTAAAAC
Reverse Primer position: 872-893
Reverse Primer GC number: 9
Reverse Primer GC%: 40.909090909090914
Reverse Primer length: 22
Primer length difference: 1
Forward Primer GC clamp: Yes
Reverse Primer GC clamp: Yes
Forward Primer Tm: 62.0
Reverse Primer Tm: 62.0
tm difference: 0.0
PCR product length: 497
PCR product position: 397-893
Cross-Dimer: No
Self-Dimer(f,f): No
Self-Dimer(r,r): No
Forward Primer hairpin: No
Reverse Primer hairpin: No
Forward Primer specificity: Yes
Reverse Primer specificity: Yes
Forward Primer repeat in template DNA for specificity: 1
Reverse Primer repeat in template DNA for specificity: 1
```

Figure 18. Primer pair information retrieved from the output file.
